# Validation of Intraluminal and Intraperitoneal microdialysis in ischemic small intestine

**DOI:** 10.1186/1471-230X-13-170

**Published:** 2013-12-10

**Authors:** Lauri Pynnönen, Minna Minkkinen, Anders Perner, Sari Räty, Isto Nordback, Juhani Sand, Jyrki Tenhunen

**Affiliations:** 1 Critical Care Medicine Research Group, Department of Intensive Care Medicine, Tampere University Hospital, Tampere, Finland; 2 Department of Intensive Care, Copenhagen University Hospital, Rigshospitalet, Denmark; 3 Department of Gastroenterology and Alimentary Tract Surgery, Tampere University Hospital, Tampere, Finland; 4 Department of Surgical Sciences, Anaesthesiology and Intensive Care, Uppsala University, Akademiska Sjukhuset, ing 70 1tr, SE 751 85, Uppsala, Sweden

**Keywords:** Microdialysis, Ischemia, Lactate to pyruvate ratio, Lactate to glucose ratio, Gut, Human, Glycerol

## Abstract

**Background:**

We sought to define the sensitivity and specificity of intraperitoneal (IP) and intraluminal (IL) microdialysate metabolites in depicting ex vivo small intestinal total ischemia during GI-tract surgery. We hypothesized that IL as opposed to IP microdialysis detects small intestinal ischemia with higher sensitivity and specificity.

**Methods:**

IL and IP microdialysate lactate, pyruvate, glucose and glycerol were analysed from small intestine of pancreaticoduodenectomy patients before and after occluding the mesenteric vasculature and routine resection of a segment of small intestine. Ex vivo time sequences of microdialysate metabolites were described and ROC analyses after 0–30, 31–60, 61–90 and 91–120 minutes after the onset ischemia were calculated.

**Results:**

IL lactate to pyruvate ratio (L/P ratio) indicated ischemia after 31–60 minutes with 0.954 ROC AUC (threshold: 109) in contrast to IP L/P (ROC AUC of 0.938 after 61–90 minutes, threshold: 18). At 31–60 minutes IL glycerol concentration indicated ischemia with 0.903 ROC AUCs (thresholds: 69 μmol/l). IP glycerol was only moderately indicative for ischemia after 91–120 minutes with 0,791 ROC AUCs (threshold 122 μmol/l). After 31–60 minutes IL and IP lactate to glucose ratios (L/G ratio) indicated ischemia with 0.956 and 0,942 ROC AUCs (thresholds: 48,9 and 0.95), respectively.

**Conclusions:**

The results support the hypothesis that intraluminal application of microdialysis and metabolic parameters from the small intestinal lumen indicate onset of ischemia earlier than intraperioneal microdialysis with higher sensitivity and specificity.

## Background

Microdialysis enables the researcher or the clinician to investigate local tissue metabolism and to detect insufficient perfusion of a selected tissue [1-6]. Intestinal application of the method has been tested in several different experimental settings while clinical investigations are still scarce. Intramural [7,8], intraluminal [9-12] or intraperitoneal [1,3-6,9,11,13] location of microdialysis catheters has been suggested by different investigators.

While intramural microdialysis may be the ideal method in terms of supplying signal from the interstitium within the wall of intestine, the invasive nature of this approach limits its clinical application, as the placement of the probe causes surgical trauma and affects the local metabolism up to 1 h [9]. Either intraluminal or intraperitoneal (gut serosal) access might serve as a surrogate for metabolic signal from intestinal cells. In particular, intraluminal release of metabolic markers putatively reflects the status of intestinal epithelial cells [7]. It is reasonable to assume that metabolite release to the lumen of gut is either spill over from the deeper tissue or directly from intestinal epithelial cells.

It has been postulated that gut may serve not only as a target organ to, but indeed a source of systemic inflammation by releasing bacteria or toxins to blood or lymph (gut hypothesis). Therefore it is rational to hypothesize that the viability of intestinal epithelial cells is the logical specific target for monitoring gut epithelial barrier function [7,9,14-19].

High lactate and the lactate-to-pyruvate ratio or glycerol release to the bowel lumen are considered as early and specific markers of the damage of the gut epithelial cells [8,9,12,14]. Alternatively, the oxygenation/metabolism of the gut wall can be measured intraperitoneally (inside the abdominal cavity against the serosal surface of the gut) ((animal studies; 1, 9, 11, 13, human studies; 3, 4, 5, 6)). This approach obviously is farther from the intestinal epithelial cells and therefore might describe a different aspect of gut viability. A direct comparison of the two approaches in clinical setting is lacking.

Our purpose was to determine which localisation for the microdialysis catheter enables the monitoring of gut wall metabolism with high sensitivity and specificity to depict gut wall ischemia.

In addition, because as early signal as possible is needed to alert the clinician for the adverse event, the time sequence of the signals was analysed. To the best of our knowledge, there is no clinical report on the comparison between intraperitoneal and intraluminal microdialysis. We specifically hypothesized that intraluminal microdialysis offers earlier and more sensitive signal for ischemic gut.

## Methods

This was an observational, prospective non-interventional trial and therefore it was not listed in Clinical Trials.gov. The study was approved by the institutional ethical review board of Tampere University Hospital, Finland.

Twenty patients scheduled for elective pancreatic carcinoma surgery in the Tampere University Hospital were enrolled into this study after informed consent procedure. Inclusion criteria were the patient’ age > 17 years of age and need for pancreaticododenectomy (Whipple’s procedure). Whipple procedure patients were chosen as surgically and ethically suitable group of patients since the Whipple specimen that is removed during the operation includes a segment of (healthy) jejunum that can be used for not only pathology analyses but also for a total gut ischemia model ex vivo. Exclusion criteria were a pre-existing inflammatory bowel disease or prior enrolment to any interventional trial within 30 days prior to this study.

Anaesthesia was induced according to in house protocol by boluses of propofol (Propofol Fresenius Kabi (10 mg/ml), Fresenius Kabi Ab, GER) and Fentanyl (Fentanyl (50 μg/ml) Janssen-Cilag Oy, SWZ). Neuromuscular blockage was induced by cis-atracurium (Nimbex, (5 mg/ml) GlaxoSmithKline, UK). Anaesthesia was maintained by infusion of remifentanyl (Ultiva (100 mg/ml), GlaxoSmithKline, UK) and inhaled sevofluran (Sevorane, Abbott Scandinavia AB, SWE), the muscle relaxation was added as needed according to Train-of-Four measurement. Adequate intravascular volume and systemic arterial blood pressure was maintained using NaCl 0,9% and/or Ringer 1000 infusions and infusion of noradrenalin (Levophed (1 mg/ml) Hospira, UK) if needed.

Surgical method in brief: The same team of surgeons performed each operation (JSa, SRä, INo). Through a bilateral subcostal laparotomy, after inserting the two first microdialysis catheters into the gut lumen (intraluminal) and on the serosal surface (intraperitoneal, antemesenterial pouch was prepared by superficial serosal sutures for the catheter) of the segment down stream of ligament of Treitz in jejunum was visualized, prepared and resected as part of final Whipple specimen. While the abdominal exploration, segment visualization and preparation, the baseline samples were collected.

### Microdialysis – combination of in vivo and ex vivo validation

Two catheters, intraluminal and serosal/intraperitoneal, (CMA 62 gastrointestinal microdialysate catheter® diameter 0,6 mm, length 30 mm) were placed in the part of the jejunum that was later resected as standard procedure in Whipple operation. No additional preparations or resections were performed. Immediately following the laparotomy intraluminal microdialysis catheter was introduced to the lumen of intestine through an antemesenterical opening and sutured. The other catheter was placed on the serosal surface of the intestine and sutured. Set of superficial sutures were then applied to form a tunnel/pouch for the serosal/intraperitoneal catheter to ensure contact against serosa to mimic free placing between the intestines in intraperitoneal microdialysis. The catheters were connected to two CMA 107® pumps (CMA-Microdialysis, Stockholm, Sweden), with a flow rate of 1 μL/min. Microdialysate sampling was adjusted according to dead space in the outflow tubing and taking into account the microdialysate flow rate. Ten-minute fractions were collected over the length of the experiment, thus the aim was to collect 3 samples per patient per one thirty-minute interval after the baseline (in vivo) pre-ischemic samples. Pre-ischemic baseline samples during the surgical procedure were collected prior to clamping the vasculature of the segment. Each pre-ischemic period lasted 20–30 minutes. Therefore 2–3 baseline samples were obtained for baseline metabolic state of intestinal wall. A jejunal segment of 4–6 cm in length was removed from the Whipple specimen, before relocating the bowel and its’ mesenterium together with superior mesenteric artery and vein supply to right upper abdomen as per surgical protocol. The time of the removal of the resecate was registered, the timing of microdialysate sampling adjusted accordingly. The microdialysate samples were gathered up to 120 minutes after the closure of the vasculature of the removed bowel segment (ischemic stage/ex vivo). The resected part of small intestine was placed in a sterile cup within a warm closed water bath. The resected intestine was not in direct contact with water. The temperature was kept constant at 37–38 degrees of Centigrade during the ex vivo experimentation.

Dead space volume (with 7 minutes delay under 1 μL/min flow rate) of the microdialysate outflow tubing was accounted for by adjusting the sample collection according to events in surgery. The recovery rate of analytes to microdialysate was assumed high enough for adequate analyses. While the recovery rate can be assumed less than optimal with 1 μL/min, the decision on the flow rate was made based on the need for short (10-minute) collection time per sample and to standardized sample collection intra- and inter-individually. All the microdialysate samples were then frozen (−20°C) for later analyses. Samples were analyzed using CMA 600® Microdialysis Analyzer (CMA-Microdialysis, Stockholm, Sweden) in a batch after storage.

During the surgery arterial blood gas analyses were performed at the beginning of the surgery and at the beginning of the local ischemia (mesenteric arterial occlusion).

### Calculations and statistical analyses

All data were calculated using SPSS for Windows, version 17.0 (SPSS Inc., Chicago, IL, USA). Results are expressed as median (quartiles) unless otherwise indicated. Receiver Operating Characteristics (ROC) analysis was performed for lactate/pyruvate ratio, glycerol, and lactate/glucose ratio at four time segments of the ex vivo experiment: 0–30 minutes, 31–60 minutes, 61–90 minutes and 91–120 minutes after occluding the mesenteric vessels immediately prior to gut resection. The highest area under the curve (AUC) value and the best threshold value is given. The best threshold value is reported as the closest to (0,1) point value. As the data were not normally distributed (Kolmogorov-Smirnov), in line with the descriptive statistics, non-parametric tests Mann–Whitney U and Wilcoxon’s Signed rank tests were used. P < 0.05 was considered statistically significant.

## Results

A total of 21 patients (10 females and 11 males) with the age of 66 (56–71) years were enrolled after written informed consent. One patient was excluded because technical failure of microdialysis catheters. The systemic hemodynamics and metabolic conditions were stable during to the surgery prior to the beginning of the local gut ischemia (Table 1).

**Table 1:** Hemodynamics in the beginning of the surgery and in the beginning of the ischemia

**Hemodynamics**														
	**pH**	**n**	**gluc (mmol/l)**	**n**	**Lact (mmol/l)**	**n**	**Sa (%)**	**n**	**MAP**	**n**	**pulse**	**n**	**CVP**	**n**
**In the beginning of the surgery**	7,42 (7,39–7,44)	18	6,55 (5,98–7,15)	18	0,7 (0,5–0,85)	18	99 (98–100)	20	70 (63,5–81,5)	20	68 (56–75)	20	9 (7–11)	19
**Before ischemia**	7,39 (7,38–7,46)	13	6,9 (5,93–7,53)	14	0,7 (0,6–1,28)	12	100 (99–100)	20	74 (65,75–78)	20	64 (55–70)	20	7 (7–9)	19

Values are means (quartiles).

### Lactate/pyruvate ratio

**Figure 1:**
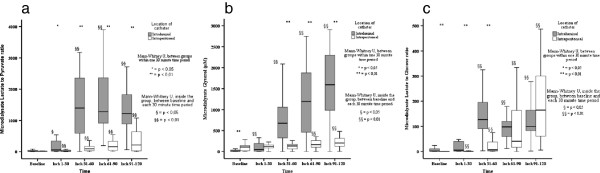
**Intraluminal and intraperitoneal microdialysate metabolite signals during the baseline and four ischemia periods. a**. Intraluminal and intraperitoneal lactate to pyruvate ratios during baseline and four ischemic time periods measured by microdialysis. **b**. Intraluminal and intraperitoneal glycerol (μmol/l) during baseline and four ischemic time periods measured by microdialysis. **c**. Intraluminal and intraperitoneal lactate to glucose ratios during baseline and four ischemic time periods measured by microdialysis.

**Figure 2:**
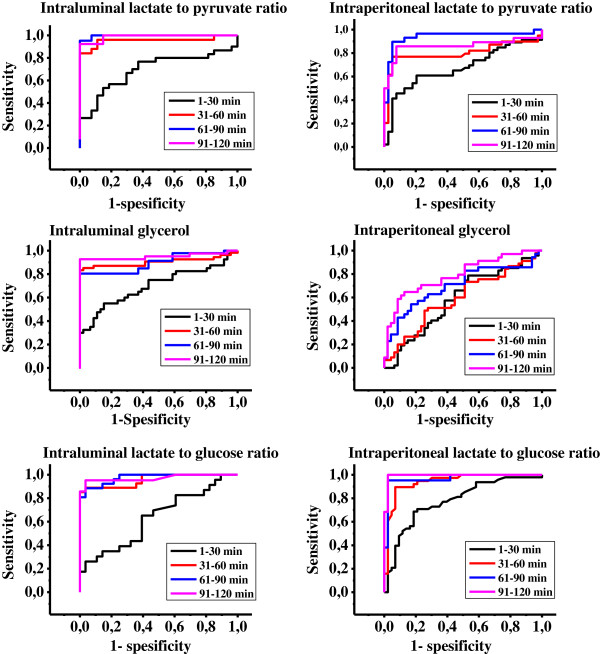
ROC- curves for intraluminal and intraperitoneal lactate to pyruvate and –glucose ratios and glycerol.

Pre ischemic L/P- ratio was 16 (10–51) intraluminally and 14 (from 8–18) intraperitoneally with lactate concentrations of 0.1 (0.03- 0.4) mM and 0.7 (0.2– 1.5) mM, respectively. Intraluminal microdialysate L/P ratio increased to its maximum of 88-fold (at 31–60 minutes) in comparison to maximum of 15-fold increase intraperitoneally (91-120 minutes) during the 120 minutes of observation (Figure 1a). Intraluminal L/P ratio indicated gut ischemia after 31–60 minutes after onset of ischemia (ROC AUC of 0.954) with 96% sensitivity and 89% specificity with the threshold L/P ratio of 109. Intraperitoneal L/P ratio indicated gut ischemia at 61–90 minutes after onset of ischemia (ROC AUC of 0.938) with 97% sensitivity and 80% specificity with threshold L/P ratio of 18 (Figure 2 and Table 2).

**Table 2:** Intraluminal and Intraperitoneal sensitivity, specificity, threshold and AUC - values for detecting ischemia in human gut by lactate to pyruvate ratio, glycerol and lactate to glucose ratio

	**Intraluminal**					**Intraperitoneal**			
Minutes after ischemia					Minutes after ischemia				
1–30	Threshold level	Sensitivity	Specificity	AUC*	1–30	Threshold level	Sensitivity	Specificity	AUC*
	18.95	73.30%	63%						
L/P	22.35	70.00%	66.70%		L/P	15.37	63.00%	56.40%	
	28.79	66.70%	66.70%	0.695		18.59	60.90%	79.50%	0.673
						19.54	58.70%	79.50%	
Glycerol μmol/l	19.78	67.50%	60.90%		Glycerol μmol/l	100.61	68.10%	48.90%	
	20.07	65.00%	60.90%	0.701		108.70	66.00%	55.30%	0.584
	20.49	65.00%	63.00%			114.73	57.40%	57.40%	
L/G	1.75	73.90%	39.30%		L/G	0.38	70.80%	79.10%	
	3.75	60.90%	60.70%	0.634		0.41	68.80%	81.40%	0.78
	7.59	43.50%	67.90%			0.44	64.60%	81.40%	
Minutes after ischemia					Minutes after ischemia				
31–60	Threshold level	Sensitivity	Specificity	AUC	31–60	Threshold level	Sensitivity	Specificity	AUC
L/P	68.49	96.00%	85.20%		L/P	31.27	76.90%	92.30%	
	109.42	96.00%	88.90%	0.954		35.95	76.90%	94.90%	0.800
	137.51	92.00%	88.90%			37.72	74.40%	94.90%	
Glycerol μmol/l	58.27	87.00%	89.10%		Glycerol μmol/l	103.22	62.20%	51.10%	
	68.71	87.00%	91.30%	0.903		108.92	60.00%	55.30%	0.593
	79.13	85.20%	91.30%			114.52	53.30%	57.40%	
L/G	40.50	88.90%	92.90%		L/G	0.76	89.50%	90.70%	
	48.90	88.90%	96.40%	0.956		0.95	89.50%	93.00%	0.94
	57.40	85.20%	96.40%			1.04	86.80%	93.00%	
Minutes after ischemia					Minutes after ischemia				
61–90	Threshold level	Sensitivity	Specificity	AUC	61–90	Threshold level	Sensitivity	Specificity	AUC
L/P	52.77	100.00%	81.50%		L/P	18.27	96.60%	79.50%	
	68.49	100.00%	85.20%	0.996		23.87	93.10%	87.20%	0.938
	131.40	100.00%	88.90%			31.27	89.70%	92.30%	
Glycerol μmol/l	28.82	80.40%	67.40%		Glycerol μmol/l	102.02	74.30%	48.90%	
	39.02	80.40%	80.40%	0.897		116.69	71.40%	61.70%	0.700
	58.27	80.40%	88.10%			125.57	62.90%	66.00%	
L/G	36.50	88.50%	89.30%		L/G	0.46	95.20%	81.40%	
	46.50	88.50%	96.40%	0.974		0.76	95.20%	91.70%	0.97
	58.40	80.80%	100.00%			1.97	95.20%	93%	
Minutes after ischemia					Minutes after ischemia				
91–120	Threshold level	Sensitivity	Specificity	AUC	91–120	Threshold level	Sensitivity	Specificity	AUC
L/P	27.78	100.00%	66.70%		L/P	19.11	85.70%	79.50%	
	41.13	100.00%	70.40%	0.989		27.17	85.70%	89.70%	0.860
	52.77	100.00%	81.50%			31.22	75.00%	92.30%	
Glycerol μmol/l	40.47	92.70%	82.60%		Glycerol μmol/l	115.61	76.50%	59.60%	
	49.55	92.70%	87.00%	0.950		122.43	76.50%	63.80%	0.791
	58.27	92.70%	89.10%			145.27	70.60%	76.60%	
L/G	28.50	95.20%	85.70%		L/G	0.46	100.00%	81.40%	
	36.50	95.20%	89.30%	0.971		1.97	100.00%	93.00%	0.99
	47.50	95.20%	96.40%			5	100%	97.7 %	

*AUC, area under the curve in receiver operating characteristics (ROC) analysis.

### Glycerol

Intraluminal pre-ischemic glycerol was 9 (from 4–37) μmol/l and intraperitoneal 107 (20–145) μmol/l. Ischemia increased markedly the intraluminal concentrations; intraluminal microdialysate glycerol increased to the maximum of 211-fold (at 61–90 minutes) in comparison to a 1.9-fold increase intraperitoneally (Figure 1b). Intraluminal glycerol indicated gut ischemia at 31–60 minutes after onset of ischemia (ROC AUC of 0.903) with sensitivity of 87% and specificity of 89% with threshold of 58 μmol/l. In the last time segment (91–120 minutes after onset of ischemia) ischemia was authenticated (ROC AUC of 0.950) with sensitivity of 93% and specificity of 87% by threshold of 50 μmol/l intraluminally. Intraperitoneal glycerol was moderately indicative for ischemia (ROC AUC of 0.791); intraperitoneal threshold level was in all the time segments comparable (109–122 μmol/l) and maximum sensitivity and specificity were below 77% (Figure 2 and Table 2).

### Lactate/glucose ratio

Baseline intraluminal L/G ratio was 2 (from 1 to 12) and intraperitoneal 0.2 (from 0.1 to 0.4). During ischemia, L/G- ratio increased higher intraperitonally than intraluminally. Maximum intraperitoneal increase of 863-fold occurred during 90–120 minutes after onset of ischemia. The maximum increase intraluminally was 63-fold during the second ischemic time segment, between 31–60 minutes after onset of ischemia (Figure 1c).

Metabolic signal by L/G ratio from both locations indicated ischemia after 30 minutes; Intraluminally (ROC AUC 0.956) sensitivity was 89% and specificity of 96% with threshold of 49 and intraperitoneally (ROC AUC 0.942) sensitivity was 90% and specificity of 93% with threshold of 0.95. Specificity was in both locations in all the last three time periods (31–60 minutes, 61–90 minutes and 91–120 minutes) between 88 to 100% and sensitivity between 89 to 100% (Figure 2 and Table 2).

## Discussion

The main finding of the present prospective study was that ischemia was detectable 31–60 minutes after the onset of ischemia with high ROC AUC (high sensitivity and specificity) for all three metabolic endpoints intraluminally. Intraperitoneal microdialysate metabolic parameters indicated ischemia either with lower ROC AUC (lower sensitivity and/or specificity) or later as compared to intraluminal location.

This is to our knowledge the first clinical trial where intraluminal and intraperitoneal microdialysis has been directly compared. In addition, this is the first trial to report sensitivity and specificity (ROC analysis) of each metabolic signal to depict gut ischemia. Intraluminal microdialysate metabolites reflected total gut ischemia earlier and with higher sensitivity and specificity herein. On the other hand, this was an ex vivo test of total gut ischemia. In clinical setting one may speculate if there is a need to monitor potential local ischemia as opposed to global intestinal ischemia. It is reasonable to speculate that intraluminal microdialysis sampling reflects topical metabolism of the gut wall while intraperitoneal microdialysis may reflect intestinal perfusion and metabolism from further away from the position of the catheter [1,5,6].

### Lactate to pyruvate ratio

Normal intraperitoneal L/P ratio in patients after non-complicated gastrointestinal surgery is known to be under 15 [3] and still regarded as normal when under 20 whereas the ratio being over 20 depicts hypoxia in both splanchnic [4] and central nervous system [20]. Herein intraperitoneal L/P ratios were comparable. To the best of our knowledge this is the first clinical study to report normal small intestinal intraluminal L/P ratio.

Intraluminal L/P ratio increased earlier and with higher ROC AUC as opposed to intraperitoneal location. Intraluminal L/P ratio can be affected by hypermetabolism [1,5] and severe systemic lactatemia [14], and has to be taken under consideration when interpreting the microdialysis results if studied in patients. On the other hand, intraluminal lactate is not confounded by systemic hyperlactatemia unless severe [14]. Systemic arterial hyperlactatemia on the other hand is a late sign of intestinal ischaemia as the hepatic lactate uptake increases in response to hepatic lactate influx [1]. Previously a close correlation of ischemia induced increased intestinal permeability and gut luminal lactate concentration has been proven [8].

### Glycerol

In the present study intraperitoneal baseline glycerol was of the same magnitude as normal values determined on patients after non-complicated gastrointestinal surgery, 100 μM [3,5]. Human small intestinal luminal glycerol has not been reported previously. In experimental ischemia-reperfusion both luminal and intraperitoneal glycerol increase denotes ischemia [11].

Glycerol is released when degradation of the double layer phospholipid membrane due to cell damage occurs. The damage of the epithelium in the bowel wall caused by ischemia has been studied microscopically [11] and by microdialysis [7,9-11] using glycerol as a marker of the damage and there is a discussion of glycerol potentially being even better biomarker for detecting ischemia compared to lactate. In addition to ischemia and trauma, anesthetics can affect the glycerol levels as well. Propofol fat emulsion contains glycerol and halothane is known to increase lipolysis and plasma levels of glycerol, lactate and glucose [1], thus having to take under consideration when interpreting the microdialysis results. In the present study propofol was used for the induction of anesthesia.

Glycerol release from the gut epithelial cells in total intestinal ischemia has been proven to be detectable using microdialysis either in the intestinal wall or lumen [8-12] and in the peritoneum [1,3,5]. Lumen has been suggested to be preferred place for microdialysate catheter [11]. Experimental intestinal ischemia does not alter the arterial glycerol levels [11].

In a porcine model of intestinal ischemia the steady state (non ischemic) intraperitoneal glycerol concentrations were much higher and it took 100 min from the ignition of ischemia for the increase of glycerol levels to be statistically significant when compared to non-ischemic values [9]. In this study the intraperitoneal glycerol levels were not affected by ischemia to large extent.

### Lactate to glucose ratio

Normal values for glucose varies depending of the study and study model (human/animal). Normal mean value for intraperitoneal glucose studied on humans after non-complicated gastrointestinal surgery is around 8 mM [3]. In porcine studies levels are lower; 0.91 [7], 2.05 mM [13] 3.2 mM [9] and 4.0 mM [8]. In the resting intestine (intraluminal) glucose has not been detected suggesting the level is extremely low [9]. Normal human intestinal (colon) luminal glucose has been previously reported in one trial [21]. Luminal glucose varied between 0 and 0.5 mmol/L. Small intestinal luminal glucose or lactate to glucose ratio has not been previously described.

Ischemia causes decrease in the in the interstitial glucose level in the jejunal wall [8,9,13] and in the peritoneum [9,13] and it seems that the peritoneal levels are less affected than those in the jejunal wall [13]. As the blood flow is cut of the delivery of glucose decreases. Due to the ischemia metabolism changes from aerobic to anaerobic causing accelerated uptake of extracellular glucose decreasing the level further on to sustain the energetically inefficient anaerobic glycolysis. In clinical in vivo setting transient increase in the glucose concentration occurs possibly due to additional glucose delivery source or a drop in metabolism or compensatory autoregulatory responses as speculated by Deeba and colleagues [21].

There are limitations to our investigation. It is reasonable to assume that the state of single part of the bowel does not predict the state of another part in terms of circulation and metabolism. Heterogeneous perfusion patterns have been described earlier in different disease models. Therefore it is of necessity to develop non-invasive methods that reliably describe events in different parts of the gastrointestinal tract. On the other hand, in certain surgical procedures post-operative monitoring may be aimed at specific locations such as anastomosis area. While we state here that the localisation of the two catheters were intraluminal and intraperitoneal, it should be noted that the papers describing intraperitoneal application previously applied the catheter in such manner that the catheter collected samples of intraperitoneal fluid with the contribution of parietal peritoneum as well. In clinical scenario not only the mesenteric circulation affects the constitution of peritoneal fluid but also the uptake and release of substances by the parietal peritoneum. Herein, the catheter was enveloped between the serosal surfaces of the antemesenteric part of the small intestine. Therefore only gut serosal wall had an impact on the metabolite concentrations. Finally, we chose to use 1 microliter/min dialysate perfusion rate in order to obtain adequate sample volume per short time fragment. This may have impact to glycerol concentration obtained from the two locations in the present experiment. Therefore the absolute upper normal threshold may vary between trials. Conversely, lactate to pyruvate ratio and lactate to glucose ratio is not confounded by the dialysate perfusion rate since the recovery rate of glucose and lactate to the dialysate can be considered comparable. Thus L/P ratio and L/G ratios can be translated comparable to reports with microdialysis with other perfusion rates.

## Conclusions

The results support the hypothesis that intraluminal application of microdialysis and metabolic parameters from the small intestinal lumen indicate onset of ischemia earlier with higher sensitivity and specificity.

## Competing interests

The authors have no competing interests to declare.

## Authors’ contributions

JTE, MM, SR, JS, and IN conceived and designed the trial. JTE, MM, SR, JS and IN contributed to trial execution. LP and JTE analysed the data wrote the manuscript. All authors contributed to writing the manuscript. All authors read and approved the final manuscript.

## Pre-publication history

The pre-publication history for this paper can be accessed here:

http://www.biomedcentral.com/1471-230X/13/170/prepub
